# Emerging role of ferroptosis in glioblastoma: Therapeutic opportunities and challenges

**DOI:** 10.3389/fmolb.2022.974156

**Published:** 2022-08-17

**Authors:** Shenghua Zhuo, Guiying He, Taixue Chen, Xiang Li, Yunheng Liang, Wenkai Wu, Lingxiao Weng, Jigao Feng, Zhenzhong Gao, Kun Yang

**Affiliations:** ^1^ Department of Neurosurgery, First Affiliated Hospital of Hainan Medical University, Haikou, China; ^2^ Department of Neurology, Shenzhen Sixth People’s Hospital, Huazhong University of Science and Technology Union Shenzhen Hospital, Shenzhen, China; ^3^ Department of Neurosurgery, Second Affiliated Hospital of Hainan Medical University, Haikou, China

**Keywords:** glioblastoma, ferroptosis, cancer therapy, therapy resistance, immunotherapy

## Abstract

Glioblastoma (GBM) is the most common malignant craniocerebral tumor. The treatment of this cancer is difficult due to its high heterogeneity and immunosuppressive microenvironment. Ferroptosis is a newly found non-apoptotic regulatory cell death process that plays a vital role in a variety of brain diseases, including cerebral hemorrhage, neurodegenerative diseases, and primary or metastatic brain tumors. Recent studies have shown that targeting ferroptosis can be an effective strategy to overcome resistance to tumor therapy and immune escape mechanisms. This suggests that combining ferroptosis-based therapies with other treatments may be an effective strategy to improve the treatment of GBM. Here, we critically reviewed existing studies on the effect of ferroptosis on GBM therapies such as chemotherapy, radiotherapy, immunotherapy, and targeted therapy. In particular, this review discussed the potential of ferroptosis inducers to reverse drug resistance and enhance the sensitivity of conventional cancer therapy in combination with ferroptosis. Finally, we highlighted the therapeutic opportunities and challenges facing the clinical application of ferroptosis-based therapies in GBM. The data generated here provide new insights and directions for future research on the significance of ferroptosis-based therapies in GBM.

## 1 Introduction

Glioblastoma (GBM) is the most prevalent malignant tumor of the central nervous system, accounting for 54% of adult malignant cases (Miller et al., 2021). In the past 4 decades, limited significant strides have been made in its prevention, early detection, and treatment (Miller et al., 2021). Although several therapies have been developed for the treatment of GBM including surgery, radiation, and chemotherapy, patients with GBM have a poor prognosis, only 5.5% of patients survived 5 years post-diagnosis (Ostrom et al., 2016). GBM treatment faces significant challenges, such as blood-brain barrier (Xie et al., 2021), high intra-tumoral or inter-tumoral heterogeneity (Patel et al., 2014; Jacob et al., 2020), and immunosuppressive microenvironment (Fu et al., 2020). Nonetheless, an increasing understanding of the complex and interrelated tumor microenvironment (TME) has expanded the range of therapeutic strategies (Fu et al., 2020). So far, the outcomes of monotherapy have been disappointing therefore, combination therapy is required to achieve a broad and lasting anti-tumor response. Therefore, the present study focuses on the development of effective molecular targeted therapy, immunotherapy, gene therapy, and novel drug delivery technology.

Tumor cells resist cell death and evade immune destruction, which is different from normal tissue cells. Regulatory cell death (RCD) is a type of death initiated by gene regulation that originates from the intracellular or extracellular microenvironment when other adaptive responses cannot restore cell homeostasis. RCD can be subdivided into necroptosis, pyroptosis, ferroptosis, and other types of cell death as per its mechanism (Galluzzi et al., 2018). In 2012, researchers found lethal compounds responsible for ferroptosis, a new model of cell death. Ferroptosis is morphologically, genetically, and biochemically distinct from necrosis, apoptosis, and autophagy; it is characterized by iron dependence and reactive oxygen species (ROS) accumulation (Dixon et al., 2012). Cell morphological changes include rupture and blistering of the cell membrane; contraction and increase of membrane density; decrease or disappearance of the mitochondrial ridge; rupture of the mitochondrial outer membrane and normal size nucleus without chromatin agglutination. Under the induction of iron ions, ROS accumulation causes an imbalance of redox in cells, resulting in the occurrence of ferroptosis (Dixon et al., 2012). Iron-dependent tumor cells are vulnerable to ferroptosis inducers (FINs). Ferroptosis influences the efficacy of chemotherapy, radiotherapy, and immunotherapy, hence drug combination targeting ferroptosis signals improves the current efficacy of these treatments (Sato et al., 2018; Lang et al., 2019). Therefore, drug combination with drugs targeting the pathways of ferroptosis is a novel therapeutic strategy.

Ferroptosis is closely associated with cerebral ischemia-reperfusion injury (Tuo et al., 2022), neurodegenerative diseases (Park et al., 2021), and tumors (Dixon et al., 2012). Besides its role in acquired drug resistance and cancer immunosuppression, ferroptosis is involved in metabolic reprogramming, providing a novel opportunity for drug-resistant tumors (Tarangelo et al., 2018). Noteworthy, GBM cells have strong anti-apoptosis capacity and inhibitory tumor immune microenvironment (TIME) as an immune-desert tumor, resulting in a poor response to immunotherapy. T cells showed particularly severe exhaustion signature in GBM (Woroniecka et al., 2018). At present, GBM treatments transform “cold” tumors into “hot” tumors, hence stimulating the immune system to fight tumors (Zhang J. et al., 2021). Studies indicate that ROS levels significantly increase after anti-PD-L1 treatment, hence decreasing the sensitivity of immunotherapy after suppressing ferroptosis (Wang W. et al., 2019). Therefore, therapeutic approaches targeting ferroptosis may provide a novel and promising approach for killing GBM cells.

Recent bioinformatics studies have shown that ferroptosis-related genes (FRGs) can be used to predict the treatment response in GBM (Zhuo et al., 2020; Xiao et al., 2021; Dong et al., 2022; Tian et al., 2022; Zhang et al., 2022). Therefore, ferroptosis plays an important role in GBM. Evasion of ferroptosis may increase GBM invasiveness and development of drug resistance. Previous review has indicated that alterations in glucose, lipid, glutamine, and iron metabolism in GBM may increase sensitivity to ferroptosis due to the enhanced reliance on the antioxidant system and iron ions (Huang et al., 2021). Thus, targeting ferroptosis can be a potential treatment for GBM. This review describes the role of ferroptosis in GBM treatment, including chemotherapy, radiotherapy, immunotherapy, and targeted therapy, as well as its opportunities and challenges.

## 2 Molecular mechanisms of ferroptosis

### 2.1 Drivers of ferroptosis

**FIGURE 1:**
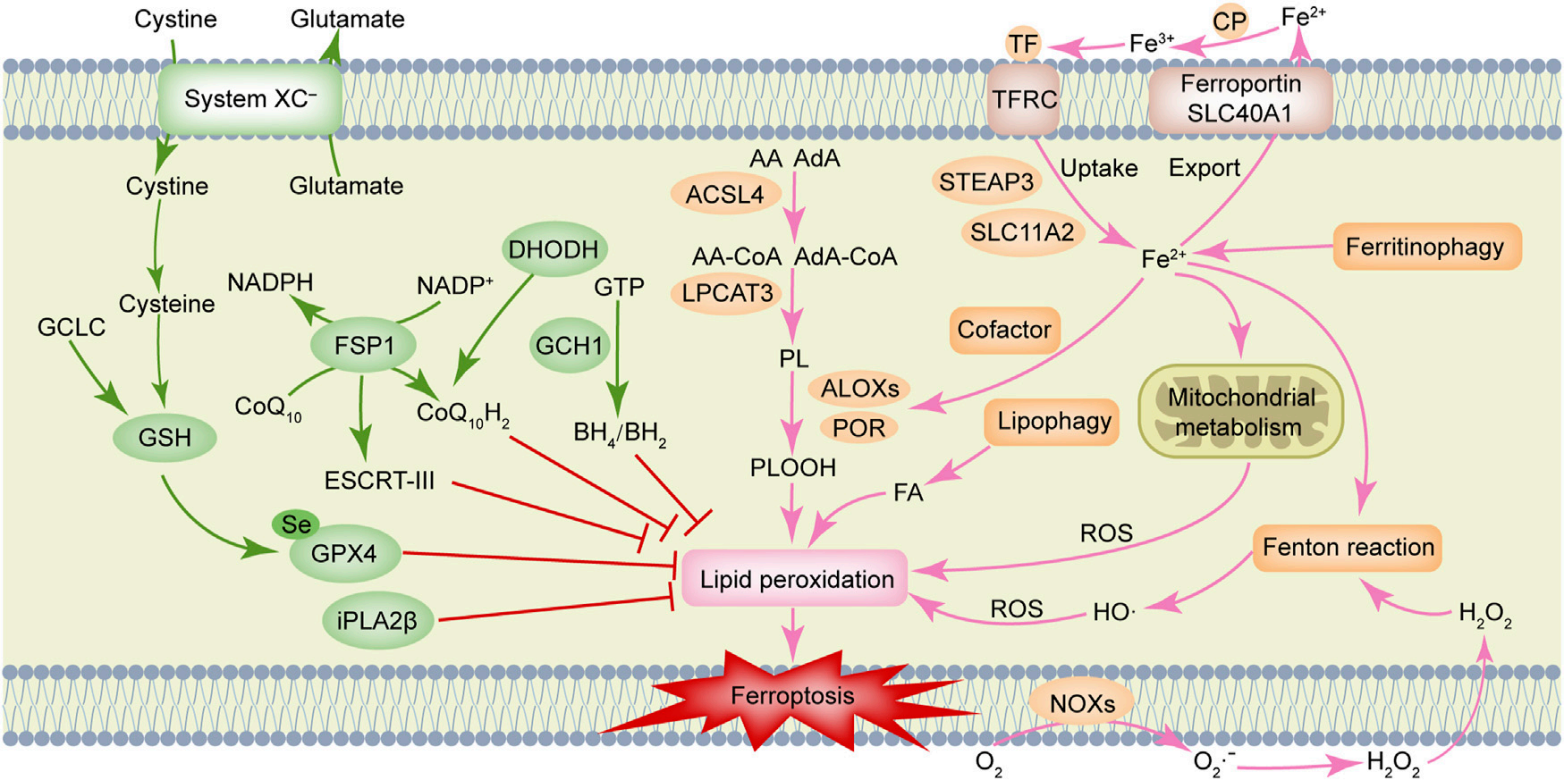
Molecular mechanisms of ferroptosis. Ferroptosis is driven by accumulation of polyunsaturated fatty acid-containing phospholipids (PUFA-PLs), transition metal iron and reactive oxygen species (ROS). Ferroptosis defense systems include cyst(e)ine/GSH/GPX4 axis, NAD(P)H/FSP1/CoQ_10_ axis, DHODH/CoQ_10_ axis, GCH1/BH_4_ axis, and iPLA2β, etc. AA, arachidonic acid; ACSL4, Acyl-coenzyme A synthetase long-chain family member 4; AdA, adrenic acid; ALOXs, arachidonate lipoxygenases; BH_2_, dihydrobiopterin; BH_4_, tetrahydrobiopterin; CoQ_10_, ubiquinone; CoQ_10_H_2_, ubiquinol; CP, ceruloplasmin; DHODH, dihydroorotate dehydrogenase; ESCRT-III, endosomal sorting complex required for transport-III; FA, fatty acid; FSP1, ferroptosis suppressor protein 1; GCH1, GTP cyclohydrolase 1; GCLC, glutamate-cysteine ligase catalytic subunit; GPX4, glutathione peroxidase 4; GSH, glutathione; GTP, guanosine triphosphate; H_2_O_2_, hydrogen peroxide; iPLA2β, calcium-independent phospholipase A2β; LPCAT3, lysophosphatidylcholine acyltransferase 3; NOXs, NADPH oxidases; PL, phospholipids; POR, cytochrome P450 oxidoreductase; SLC11A2, solute carrier family 11 member 2; SLC40A1, solute carrier family 40 member 1; STEAP3, STEAP family member 3, metalloreductase; TF, transferrin; TFRC, transferrin receptor.

Ferroptosis is driven by lethal phospholipid peroxidation resulting from imbalanced redox homeostasis and cellular metabolism. This RCD process relies on phospholipids containing polyunsaturated fatty acid chains (PUFA-PLs), transition metal iron and ROS (Figure 1). This paragraph discusses mechanisms of liposynthesis, storage, utilization, and peroxidation during ferroptosis modulation. As a key metabolic substrate for fatty acid synthesis, acetyl-CoA is mainly converted from mitochondria-derived citrate through the action of ATP citrate lyase (ACLY) (Wei et al., 2020). The rate-limiting step in fatty acid synthesis is the synthesis of malonyl-CoA from acetyl-CoA by acetyl-CoA carboxylase (ACC) (Wang et al., 2022). Then malonyl-CoA and acetyl-CoA are catalyzed and condensed by fatty acid synthase (FASN) to form 16-carbon fatty acid palmitate. After palmitate is elongated by ELOVL fatty acid elongase (ELOVL), the initial product of fatty acid synthesis is further desaturated by fatty acid desaturases. Among them, delta-5 desaturase (D5D) and delta-6 desaturase (D6D) are rate-limiting enzymes for PUFAs conversion and are considered to be the main determinants of PUFA levels (Tosi et al., 2014), whereas Stearoyl-CoA Desaturase (SCD) catalyzes the formation of monounsaturated fatty acids (MUFAs) (Ntambi, 2004). Exogenous MUFAs induce a ferroptosis-resistant cell state by decreasing levels of oxidizable PUFAs and suppressing the accumulation of lipid peroxides (Magtanong et al., 2019). Lipid droplets can buffer and store excess lipids. Increased lipid droplet degradation promotes ferroptosis (Bai et al., 2019). Fatty acids are catabolized by fatty acid beta-oxidation (FAO) in mitochondria through a series of reactions that shorten them. Carnitine palmitoyltransferase 1 (CPT1) present in mitochondrial outer membrane can catalyze carnitine esters from acyl-CoA as a rate-limiting step of FAO (van der Leij et al., 1999). Lipid peroxidation is a process in which carbon-carbon double bonds of lipids (especially PUFAs) are attacked by oxidants (such as free radicals). Two membrane remodeling enzymes, an Acyl-CoA synthetase long-chain family member 4 (ACSL4) and lysophosphatidylcholine acyltransferase 3 (LPCAT3), are key drivers of iron ptosis, as revealed by genome-wide haploid and CRISPR-Cas9 screening (Dixon et al., 2015; Doll et al., 2017). ACSL4 can catalyze the connection between CoA and long-chain PUFAs (including arachidonic acid (AA) and adrenic acid (AdA)). LPCAT3 will re-esterify these products into phospholipids (PL), increasing the cellular incorporation of long-chain PUFAs into membranes and lipids. It has been found that some arachidonate lipoxygenases (ALOXs) have the capability of directly oxygenating PUFAs and PUFA-containing lipids within biological membranes, promoting the production of phospholipid hydroperoxides (PLOOHs), a lipid-based form of ROS, thereby mediating ferroptosis (Yang et al., 2016). Furthermore, cytochrome P450 oxidoreductase (POR) (Zou et al., 2020) and NADPH oxidases (NOXs) (Dixon et al., 2012) play a role in lipid peroxidation and contribute to ferroptosis.

Iron homeostasis is an important factor that determines ferroptosis sensitivity. It has been described in detail in a prior review that ferroptosis is tightly controlled by processes associated with iron metabolism including iron uptake, storage, utilization, and efflux (Chen X. et al., 2020). Besides initiating the non-enzymatic Fenton reaction to peroxidize PUFA-PLs, iron also acts as an essential cofactor for enzymes involved in lipid peroxidation, such as POR and ALOXs, which in turn also regulate ferroptosis. Ferroptosis susceptibility can be affected by cellular labile iron content. Ferroptosis, for instance, is triggered by increased cellular iron availability as a result of autophagic degradation of ferritin, the iron storage protein (Hou et al., 2016). Similarly, transferrin receptor and glutaminolysis also contribute to ferroptosis (Gao et al., 2015).

### 2.2 Ferroptosis defense systems

Reports have been made of multiple ferroptosis defense systems (Figure 1). Among them, cyst(e)ine/GSH/GPX4 axis is the primary regulator of ferroptosis. The cystine/glutamate reverse transporter (system XC^–^), is composed of a heavy chain solute carrier family 3 members 2 (SLC3A2) and a light chain solute carrier family 7 member 11 (SLC7A11), plays a role in cysteine synthesis (Koppula et al., 2021). Cysteine is converted into glutathione (GSH) with the help of gamma-glutamylcysteine synthetase and GSH synthetase. Glutathione Peroxidase 4 (GPX4) acts as a phospholipid hydroperoxidase to convert PLOOH into phospholipid alcohol (PLOH), preventing ferroptosis from occurring (Yang et al., 2014). Inhibition of SLC7A11 prevents glutamate/cystine exchange and reduces intracellular GSH synthesis, which further inhibits the activity of GPX4, impairs intracellular antioxidant system, and triggers ferroptosis (Koppula et al., 2021). Notably, erastin (SLC7A11 inhibitor) and RSL3 (GPX4 inhibitor) can induce ferroptosis (Dixon et al., 2012; Yang et al., 2014). The ubiquinone (CoQ_10_) oxidoreductase ferroptosis suppressor protein 1 (FSP1) is a glutathione-independent ferroptosis suppressor. FSP1 can directly reduce lipid radicals and terminate lipid peroxidation by reducing CoQ_10_ to ubiquinol (CoQ_10_H_2_, the reduced and active antioxidant form of CoQ_10_) and/or regenerating oxidized alpha-tocopherol radical (vitamin E) to its non-radical form (Bersuker et al., 2019; Doll et al., 2019). In some cases, the membrane repair mechanisms of ESCRT-III (a protein complex) may also allow FSP1 to inhibit ferroptosis in a pathway parallel to that of CoQ_10_H_2_ (Dai et al., 2020). Further, a recent study revealed that dihydroorotate dehydrogenase (DHODH) is located in the inner mitochondrial membrane and operates in parallel with mitochondrial GPX4 (but independent of cytosolic FSP1 or GPX4) to inhibit ferroptosis by reducing CoQ_10_ to CoQ_10_H_2_ (Mao et al., 2021).

In another study, it was reported the metabolic products tetrahydrobiopterin (BH_4_) and dihydrobiopterin (BH_2_) derived by GTP cyclohydrolase 1 (GCH1) were shown to protect against ferroptosis by acting as a direct radical-trapping antioxidant and being involved in CoQ10 synthesis (Kraft et al., 2020). Recent studies indicated that calcium-independent phospholipase A2β (iPLA2β) preferentially hydrolyzes peroxidized PUFA-PLs and that it represses ferroptosis induced by p53 in a GPX4-independent manner (Chen D. et al., 2021; Sun et al., 2021). As summarized in the previous review, oxidative-stress-responsive transcription factor nuclear factor erythroid 2-related factor 2 (NRF2) can mitigate ferroptosis by stimulating the expression of many of its canonical target genes (Anandhan et al., 2020). Additionally, the accumulation of squalene (a metabolite of the cholesterol pathway) has been reported to have anti-ferroptotic effects in cholesterol auxotrophic lymphoma cell lines and primary tumors (Garcia-Bermudez et al., 2019).

## 3 The role of ferroptosis in GBM treatment

### 3.1 Chemotherapy

One of the major reasons for cancer treatment failure is the resistance of malignant tumor cells to chemotherapeutic drugs. Unlike apoptosis, ferroptosis, a special cell death process resolves the inefficiency of apoptosis-inducing drugs. The use of FINs provides a new approach to addressing drug resistance to tumor chemotherapeutic drugs. The integrated use of FINs and chemotherapy yields a synergistic response and improves cancer sensitivity to chemotherapeutic drugs. For instance, the combination of cisplatin and erastin significantly improves anti-tumor activity, indicating the significance of ferroptosis in tumor treatment (Sato et al., 2018). Additionally, GPX4 inhibitors show a certain degree of lethality in drug-resistant cells via ferroptosis, and targeting GPX4 could be a therapeutic approach, preventing acquired drug resistance (Hangauer et al., 2017).

Recent studies have shown that the balance of oxidation and antioxidation, including ROS and GSH, is linked to its resistance to temozolomide (TMZ) treatment for GBM (Zhu et al., 2018; Wu et al., 2020). High expression of SLC7A11, a subunit of the glutamate/cystine transporter is related to poor GBM prognosis (Robert et al., 2015). TMZ increases GSH synthesis and decreases ROS levels by improving SLC7A11 expression. The combination therapy of TMZ and SLC7A11 inhibitor erastin are potentially effective GBM treatments (Chen et al., 2015). Sulfasalazine (SAS), another SLC7A11 inhibitor causes ferroptosis in GBM cells (Sehm et al., 2016). The effect of SAS on the survival of glioma cells does not seem to depend on significant changes in autophagy, different from the cell death pathway induced by TMZ (Sehm et al., 2016). This indicates that their combination has a synergistic effect. Induction of ferroptosis is potentially one of the promising therapies against TMZ resistance. One study revealed that inhibiting autophagy causes ferroptosis and improves the sensitivity of glioblastoma stem cells (GSCs) to TMZ (Buccarelli et al., 2018). Elsewhere, GPX4 is significant in tumor resistance. One previous study reported that highly mesenchymal therapy-resistant cancer cells depend on GPX4 for survival and GPX4 function loss causes ferroptosis in these cells. This suggests that targeting GPX4 induce ferroptosis in drug-resistant cells, thereby improving their sensitivity to chemotherapy medication (Hangauer et al., 2017). Considering that RSL3 inhibits GPX4 activity, the use of RSL3 improves ferroptosis in GBM cells (Fan et al., 2017; Li et al., 2021). The CRISPR-based genome-wide genetic screening and microarray analysis of ferroptosis-resistant cell lines revealed that ACSL4 dictates ferroptosis sensitivity as an essential component of ferroptosis execution by shaping cellular lipid composition (Doll et al., 2017). Additionally, ACSL4 is linked to sorafenib resistance in liver cancer (Lu et al., 2022). ACSL4 suppresses glioma cell proliferation by activating ferroptosis (Cheng et al., 2020). Moreover, ACSL4 is linked to TMZ chemosensitivity in GBM cells (Bao et al., 2021). Furthermore, NRF2 (Fan et al., 2017; Zhang and Wang, 2017) and oxidative metabolism driver activating transcription factor 4 (ATF4) (Chen et al., 2017a; Chen et al., 2017b; Gao et al., 2021) and tumor protein P53 (P53) (Blough et al., 2011; Jiang et al., 2015; Tarangelo et al., 2018) are associated with ferroptosis and TMZ resistance. Therefore, ferroptosis is closely correlated to GBM chemotherapy and significantly promotes TMZ resistance. A better understanding of the ferroptotic mechanism in TMZ resistance may provide new insights and targets in the clinical reversal of GBM.

### 3.2 Radiotherapy

Recent studies have found that radiotherapy directly causes ferroptosis in cancer cells (Lang et al., 2019; Lei et al., 2020). Cells exposed to ionizing radiation (IR) activate ROS-generating oxidases, regulate antioxidants, and disrupt metabolic activity in response to oxidative damage, thereby influencing mitochondrial function (Yang P. et al., 2021). A previous review summarized that during radiation exposure and tumor microenvironment, different types of cell death occur in irradiated tumor cells due to several factors, including cell type, oxygen tension, DNA repair capacity, P53 status, radiation dose, quality, and cell cycle stage (Sia et al., 2020). Mechanistically, IR promotes ferroptosis by generating excess ROS to induce lipid peroxidation, and ACSL4 expression to promote PUFAs biosynthesis. Ferroptosis inhibitors, including GPX4 and SLC7A11, are expressed as an adaptive response to IR (Lei et al., 2020). Moreover, ataxia telangiectasia mutated (ATM) kinase acts upstream of p53 and regulates the DNA damage response (DDR) pathway, which is critical in resolving double-strand DNA breaks (Matsuoka et al., 2007). The expression level of SLC7A11 is lowered by IR in an ATM-dependent manner and promotes ferroptosis by suppressing SLC7A11-mediated cystine uptake and GSH synthesis (Lang et al., 2019). Since SLC7A11 expression is antagonized by radiotherapy-mediated P53 activation, GSH synthesis is inhibited, hence promoting radiotherapy-induced lipid peroxidation and ferroptosis (Lei et al., 2021). Many studies have shown that ferroptosis improves the sensitivity of multiple tumor cells to radiotherapy (Lei et al., 2020; Zhang Z. et al., 2021; Feng et al., 2021; Yuan et al., 2021). Targeting GPX4 or SLC7A11 is a ferroptosis-inducing radiosensitizing approach that improves radiotherapy-induced lipid peroxidation and ferroptosis. For instance, radiotherapy-induced GPX4 and SLC7A11 expression and ACSL4 deficiency or low expression trigger radioresistance (Lei et al., 2020; Feng et al., 2021). These studies reveal a synergy between radiotherapy and ferroptosis. Induction of ferroptosis improves radiotherapy efficacy, while its inhibition reduces radiotherapy toxicity.

Radioresistance in GBM is associated with hypoxia (Marampon et al., 2014), DDR (Carruthers et al., 2018), GSCs (Osuka et al., 2021), and fatty acid oxidation (Jiang et al., 2022). Previous research has shown that doranidazole as a radiosensitizer improves radiation-induced DDR in hypoxic GSCs in a mouse model of GBM and confers survival benefits to GSC-derived tumor-bearing mice. Meanwhile, doranidazole also causes mitochondrial dysfunction and ROS accumulation in GSCs, resulting in ferroptosis (Koike et al., 2020). Radiation-induced lipid peroxidation triggers ferroptosis, which synergistically acts with FINs in GBM (Ye et al., 2020). The system XC^–^inhibitor SAS improves radiation therapy efficacy in glioma; SAS and radiation synergistically increase DNA double-strand breaks and glioma cell death. Meanwhile, SAS integrated with gamma knife radiosurgery provides a survival benefit in human GBM xenografted rats. Thus, SAS potentially acts as a radiosensitizer to improve radiotherapy efficacy in glioma patients (Sleire et al., 2015). SAS has been clinically used as a monotherapy for GBM (NCT01577966) (Robert et al., 2015) and in combination with radiosurgery (NCT04205357). Thus, these findings indicate that exploring the integrated therapeutic approach of radiotherapy and targeting ferroptosis will resolve the radiation resistance in GBM.

### 3.3 Immunotherapy

Immunogenic cell death (ICD), a cell death process that induces an immune response, allows the release or exposure of intracellular molecules from dead or dying cells and stimulates adaptive immunity, which promotes immune responses against intracellular pathogens and tumor-associated antigens (Galluzzi et al., 2018). Cell death is an integral component of an immune response, and the type and activity of damage-associated molecular patterns (DAMPs) released during ICD elicit an immune response (Galluzzi et al., 2018). A previous review noted that cell death including necroptosis, pyroptosis, and ferroptosis causes the release of DAMPs and these 3 cell death forms are potentially new mechanisms of ICD; there is an interplay between antitumor immune activation (Tang et al., 2020). As a DAMP, high mobility group box 1 protein (HMGB1) is a crucial protein necessary for the immunogenicity of cancer cells. HMGB1 binds to toll-like receptor 4 (TLR4) on DC cells, accelerating phagocytosis of DC cells as well as process and promoting antigen presentation to T cells (Yamazaki et al., 2014). FINs cause HMGB1 release in cancer cells and non-cancer cells (Wen et al., 2019). The cell death stage is crucial in the immunogenicity of ferroptotic cancer cells, and early ferroptotic cancer cells undergo ICD, accompanied by adenosine triphosphate and HMGB1 release, which stimulates bone marrow-derived dendritic cell maturation to exert anti-tumor immunity (Efimova et al., 2020). These results have narrowed the distance between ferroptosis and anti-tumor immunotherapy, laying a theoretical reference for the synergistic treatment of malignant tumors with ferroptosis and immunotherapy.

Tumor cells evade immune surveillance through various strategies, and the primary obstacle to effective antitumor immunity is highly heterogeneous, immunosuppressive, and metabolically stressful TME. Understanding the dynamic functional interactions in this intricate microenvironmental system comprising multiple immune cells, stromal cells, vascular networks, and acellular components provides vital insights into the design of precise anticancer combinatorial strategies. The capacity of iron to regulate antitumor immune response is closely linked to its significant role in tumor development (Sottile et al., 2019; Song et al., 2021). Ferroptosis modulate immune cells in the TME and in crosstalk between tumor and immune cells, which are new insights into targeting ferroptosis in cancer immunotherapy (Wang W. et al., 2019; Lang et al., 2019; Ma et al., 2021). Interferon Gamma (IFN-γ) secreted by cytotoxic CD8^+^ T cells downregulates the expression of system XC^–^, sensitizing cancer cells to ferroptosis. PD-L1 antibodies and FINs synergistically suppress tumor growth *in vitro* and *in vivo*, and melanoma patients with clinical benefit from immunotherapy express a genetic signature of T-cell-induced ferroptosis, highlighting the potential of targeting the ferroptosis pathway to improve cancer immunotherapy (Wang W. et al., 2019). IFN-γ derived from immunotherapy-activated CD8^+^ T cells synergizes with radiotherapy-activated ATM to cause ferroptosis in cancer cells (Lang et al., 2019). Importantly, CD36 mediates fatty acid uptake by CD8^+^ T cells, causes lipid peroxidation and ferroptosis, and reduces cytotoxic factor production, impairing CD8^+^ T antitumor function (Ma et al., 2021). Targeting CD36 or inducing ferroptosis improves CD8^+^ T efficacy of cellular and immune checkpoint blockade-based tumor immunotherapy (Ma et al., 2021). Conditional deletion of Gpx4 induces ferroptosis in T cells by lipid peroxidation in mice (Matsushita et al., 2015). The fate of tumor cells appears to be determined by whether tumor cells and tumor suppressor immune cells coexist, as well as the sequence of ferroptosis. On the one hand, ferroptosis of tumor cells produce DAMPs and promote an immune response. Conversely, tumor-suppressing immune cells undergo ferroptosis, whereas tumor cells escape death. Therefore, in-depth studies of these crosstalk relationships are necessary to elucidate the role of ferroptosis as an ICD or inhibition of tumor suppressor immune cells in inducing or inhibiting immune responses.

As a low-immunogenic tumor, GBM has numerous immunosuppressive mechanisms such as low mutational burden (Hodges et al., 2017) and immunosuppressive microenvironment (Fu et al., 2020). In mouse glioma cells, ferroptosis inhibitors target photodynamic therapy-induced ICD (Turubanova et al., 2019). Relevant studies based on public databases indicate that risk scores based on the ferroptosis-related genes predict prognosis and immunotherapy response in GBM (Zhuo et al., 2020; Xiao et al., 2021). Stimulator of interferon genes (STING) is crucial for promoting anti-tumor immune responses against cancer (Mender et al., 2020). GPX4 promotes STING activation by maintaining lipid redox homeostasis (Jia et al., 2020). STING promotes anti-glioma immunity by causing type I IFN signaling (Ohkuri et al., 2014). RSL3 exerts antitumor effects via NF-κB pathway activation and GPX4 depletion driving ferroptosis in GBM (Li et al., 2021). These findings indicate that GPX4, a key ferroptotic gene, is closely related to an immune response in the TME, and its role in GBM antitumor immunotherapy remains uninvestigated.

In the initial GBM microenvironment, glioma-associated microglia/macrophages (GAMs) account for 59% of the total TME cells (Fu et al., 2020). Through symbiosis with GBM cells, GAMs regulate GSCs stemness (Shi et al., 2017), angiogenesis (Wei et al., 2021), and T cell activity (Takenaka et al., 2019). ICD is caused by ferroptosis, which polarizes tumor-promoting M2 type tumor-associated macrophages (TAMs) into anti-tumor M1 type TAMs, changes the immunosuppressive microenvironment, and enables synergistic effects of ferroptosis and immune regulation (Li and Rong, 2020; Wan et al., 2020). Elsewhere, one study revealed that ferroptosis, a predominant type of programmed cell death in gliomas, is linked to poor prognosis and immunosuppression in gliomas. Ferroptosis promotes the recruitment and polarization of TAMs to an M2-like phenotype, whereas inhibition of ferroptosis improves the sensitivity of mouse GBM to anti-PD1/L1 immunotherapy (Liu et al., 2022). A previous review summarized that cancer-associated fibroblasts (CAFs), as a vital component of TME stromal cells, modulate solid tumor growth, metastasis, immunosuppression, and drug resistance, and are linked to poor prognosis (Chen and Song, 2019). In gliomas, high expression of CAFs is linked to poor prognosis, and a risk model constructed by CAFs-related genes predicts immunotherapy response (Chen Z. et al., 2021). Exosome-like nanovesicle tumor vaccines (eNVs) targeting fibroblast activation protein-α (FAP)-positive CAFs cause-specific cytotoxic T lymphocyte immune responses that release IFN-γ and deplete FAP^+^ CAF to promote tumor ferroptosis. RSL3 improves eNVs-FAP-induced antitumor effects (Hu et al., 2021). Extensive tumor necrosis predicts a poor prognosis in GBM, and neutrophils trigger ferroptosis in GBM cells by transferring myeloperoxidase, thereby resulting in further necrosis and malignant progression of GBM (Yee et al., 2020).

In summary, the different compositions between tumor cells and immune cells in the TME could exert a certain effect on the response to immunotherapy. The relationship between ferroptosis and TIME in GBM is complex, rather than resulting in outright positive or negative effects. Furthermore, ICDs should have a balanced combination of adjuvant (DAMP-related effects) and antigenic (mainly due to tumor antigens) to induce effective antitumor immunity. In highly heterogeneous GBM, ferroptotic cells could exhibit different roles by releasing different “find me” and “eat me” signals. Therefore, investigating the molecular mechanism of ferroptosis to improve the efficacy of GBM immunotherapy is problematic.

### 3.4 Targeted therapy

The WHO 2021 classification of the central nervous system (CNS) tumors highlights the role of molecular features in the diagnosis of adult diffuse gliomas, which are important for individualized treatment and clinical prognosis of gliomas. These molecular features include isocitrate dehydrogenase (IDH) mutation status, 1p/19q co-deletion, O-6-Methylguanine-DNA Methyltransferase (MGMT) promoter methylation status, telomerase reverse transcriptase (TERT) promoter mutation, and epidermal growth factor receptor (EGFR) amplification, among others (Louis et al., 2021). A clear path to precise glioma-targeted therapy can be found by combining WHO grading, histology, and molecular characterization. Nonetheless, as a result of complex regulatory networks, classical targets such as EGFR gene alteration have failed (NCT01480479) (Weller et al., 2017). The mechanism of making targeted therapy an ideal weapon for personalized and precision medicine for GBM patients is a matter of concern. Studies indicate that targeting truncal alterations/mutations in GBM provide the greatest efficacy, providing new information for selecting GBM-targeted therapy (Lee et al., 2017). Cancer cells are therapeutically vulnerable to ferroptosis due to altered metabolic profiles, genetic mutations, and an imbalance in the ferroptotic defense system (Dixon et al., 2012). A previous review concluded that targeting ferroptosis as an anticancer strategy was effective and potential, as demonstrated by several clinical trials and preclinical drugs (Wang et al., 2021). Additionally, the mechanism of action of various targeted drugs is linked to ferroptosis, such as sorafenib (Lu et al., 2022), neratinib (Nagpal et al., 2019), and APR-246 (Birsen et al., 2022). This section describes the relationship of ferroptosis to classical therapeutic targets in GBM, including EGFR and IDH mutations.

GBM is characterized by a high frequency of EGFR amplification and/or mutation and EGFRvⅢ mutation is the most common extracellular region mutation. In contrast with wild-type EGFR, EGFRvⅢ is a more stable constitutively activated receptor (Brennan et al., 2013). EGFR/EGFRvⅢ regulates the occurrence and development of GBM by activating downstream signaling pathways, affecting GBM invasion (Micallef et al., 2009) and angiogenesis (Bonavia et al., 2012). A recent study discovered that EGFR mutants in GBM alter its function of distinguishing between different ligands and transducing biased signals by changing the extracellular structure, suggesting a new direction for the development of EGFR inhibitors (Hu C. et al., 2022). EGFRvⅢ GBM growth is dependent on lipogenesis (Guo et al., 2011). The use of fatty acid synthase inhibitors targets *in vivo* tumor growth in EGFRvⅢ GBM (Guo et al., 2009b). Additionally, the AMP-activated protein kinase (AMPK) regulates cellular energy metabolism, linking growth factor receptor signaling to cellular energy status; its activation inhibits the growth of EGFRvⅢ-expressing GBMs by targeting adipogenesis (Guo et al., 2009a). EGFR-mutated cancer cells, on the other hand, are cystine-dependent, and ferroptosis can be induced in EGFR-mutated human breast epithelial cells and non-small cell lung cancer cells after cystine deprivation (Poursaitidis et al., 2017). EGFR inhibitors including gefitinib (Song et al., 2020), erlotinib (You et al., 2021), and imatinib (Ishida et al., 2021), are associated with ferroptosis. Additionally, cetuximab, an IgG1-type human/mouse chimeric monoclonal antibody targeting the extracellular region of EGFR is closely associated with ferroptosis (Chen P. et al., 2020; Yang J. et al., 2021). Transcriptomic and genomic analyses in GBM cells with mutated activating EGFR demonstrate a range of novel resistance mechanisms, including ferroptosis and oxidative stress (Kadioglu et al., 2021). These findings suggest that treatment with ferroptosis may be more effective in overcoming the current therapeutic dilemma in GBM with EGFR amplification/mutation activation, particularly EGFRvIII.

IDH mutation status is a critical diagnostic marker for adult diffuse glioma (Louis et al., 2021). Targeting IDH mutations has certain therapeutic potential in IDH-mutant gliomas or other tumors, and various IDH mutation inhibitors have been developed. Among them, the FDA has approved enasidenib and ivosidenib (Karpel-Massler et al., 2019). Based on a previous review, multiple clinical trials have demonstrated that the IDH1-mutant small molecule inhibitor ivosidenib is biologically active and well-tolerated in patients with hematological and solid IDH1-mutant malignancies (Zarei et al., 2022). Also, the application of ivosidenib demonstrated a therapeutic effect in IDH1-mutant low-grade glioma and recurrent GBM (Mellinghoff et al., 2020; Tejera et al., 2020). A drug-transcriptome-based analysis reveals a signature of ferroptotic genes enriched in IDH-mutated brain tumors, indicating that IDH-mutated brain tumors may be uniquely vulnerable to FINs (Yang et al., 2020). IDH1 mutation improves erastin-induced lipid ROS accumulation and glutathione depletion, and its metabolite 2-Hydroxyglutarate (2-HG) sensitizes cells to ferroptosis (Wang T. X. et al., 2019). In IDH1-mutant gliomas, 2-HG inhibits glutamate levels, rendering GSH synthesis more dependent on glutaminase; suppressing glutaminase specifically improves the response of IDH-mutant glioma cells to oxidative stress and radiation sensitivity (McBrayer et al., 2018). Collectively, these studies suggest that targeting IDH mutations and inducing ferroptosis could be an effective therapeutic strategy.

## 4 Opportunities for ferroptosis in GBM treatment

### 4.1 The therapeutic potential of ferroptosis modulated by natural compounds

**FIGURE 2:**
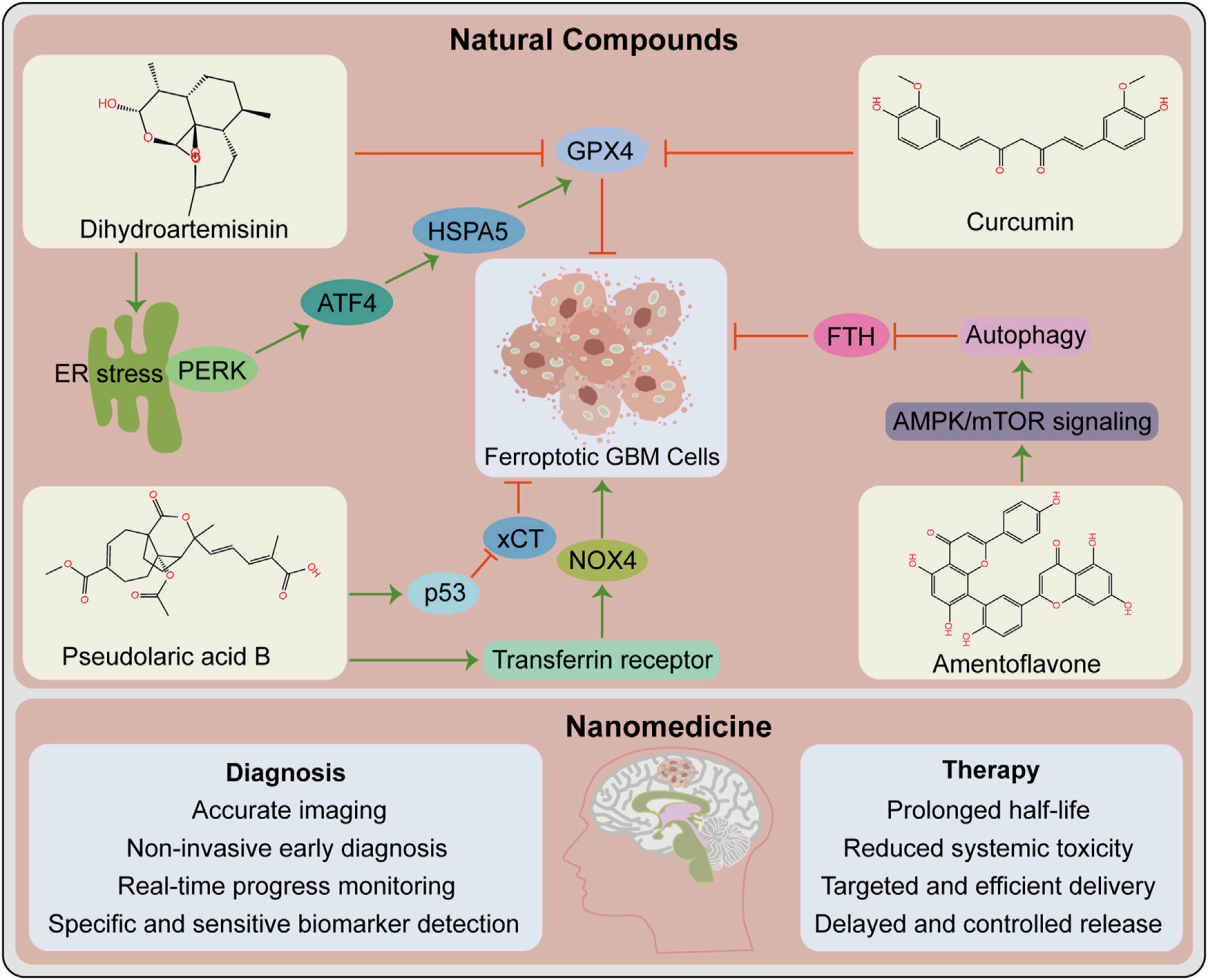
Opportunities for ferroptosis in glioblastoma therapy. Contribution of natural compounds to the treatment of glioblastoma such as dihydroartemisinin, curcumin, pseudolaric acid B and amentoflavone. The upper box shows the chemical structures of these four compounds and the associated mechanisms that regulate ferroptosis in glioblastoma. The below box lists the potential advantages of nanomedicine in the diagnosis and therapy of glioblastoma. ATF4, activating transcription factor 4; ER, endoplasmic reticulum; FTH, ferritin heavy chain. GPX4, glutathione peroxidase 4; HSPA5, heat shock protein family A (Hsp70) member 5; NOX4, NADPH oxidase 4; PERK, protein kinase R-like ER kinase; xCT, SLC7A11, solute carrier family 7 member 11.

With the advancement of research on ferroptosis-related drugs, various natural compounds have been discovered to induce ferroptosis. Some review articles summarize the roles of various natural compounds in the regulation of ferroptosis (Zhang S. et al., 2021; Ge et al., 2022). Artemisinin and its derivatives extracted from *Artemisia annua* are terpenoids that cause ferroptosis in cancer patients via various mechanisms including iron-related gene expression regulation, increased intracellular iron levels, promotion of ROS production, and intracellular GSH depletion (Ooko et al., 2015; Efferth, 2017; Hu Y. et al., 2022). Interestingly, the artemisinin derivative dihydroartemisinin causes ferroptosis in GBM cells (Chen et al., 2019; Yi et al., 2020). The phenolic compound curcumin has antioxidant and antitumor properties, exerting anti-ferroptotic or pro-ferroptotic activity in different diseases or conditions (Zhang S. et al., 2021). The curcumin analog ALZ003 induces ferroptosis in GBM cells by disrupting GPX4-mediated redox homeostasis (Chen T. C. et al., 2020). Additionally, pseudolaric acid B (diterpene acid from Cortex Pseudolaricis) (Wang et al., 2018) and amentoflavone (Chen Y. et al., 2020) (a polyphenol from Selaginella) have been confirmed *in vitro* and *in vivo* to cause ferroptosis in GBM and suppress tumor growth (Figure 2). These natural compounds have demonstrated significant therapeutic potential by causing ferroptosis in GBM. Future studies should investigate whether these natural compounds synergize with chemoradiotherapy and overcome resistance. Whether they also promote the effect of immunotherapy and targeted therapy warrants additional investigation. It is undeniable that these natural compounds may act via other pathways than ferroptosis in the treatment of GBM.

### 4.2 Application of nanomedicine in ferroptosis detection and treatment

The current traditional treatment methods have certain shortcomings in cancer treatment. With the advancement of science and technology, nanomedicine has enabled precise cancer treatment. Nanomedicine and delivery systems based on nanotechnology have numerous benefits, including high targeting efficiency, low systemic toxicity, and long half-life (Klochkov et al., 2021). Many antitumor nano-drug delivery systems have recently been developed, including strategies for causing ferroptosis and eliciting effective antitumor responses (Fu et al., 2021; Gu et al., 2021). For instance, nanoformulations combined with ferroptosis drive many pro-inflammatory signaling pathways to activate TAMs to antitumor M1 phenotype, thereby improving antitumor capacities (Gu et al., 2021). Nanotechnology-based theranostics can simultaneously diagnose and treat patients, combining different treatment modalities to improve efficacy and safety, with a wide range of applications in GBM diagnosis, drug delivery, and treatment (Tang et al., 2019) (Figure 2). Several nanotechnology-based strategies for targeting ferroptosis have been developed in GBM and have shown significant antitumor effects (Zhang et al., 2020; Zhang Y. et al., 2021). This provides additional options for preclinical research, including nanotechnology application in developing strategies for ferroptosis combined with immunotherapy that prevent off-target effects, thereby increasing the possibility of ferroptosis in GBM treatment.

## 5 Challenges of ferroptosis in GBM treatment

### 5.1 Tumor heterogeneity and stem cell characteristics

**FIGURE 3:**
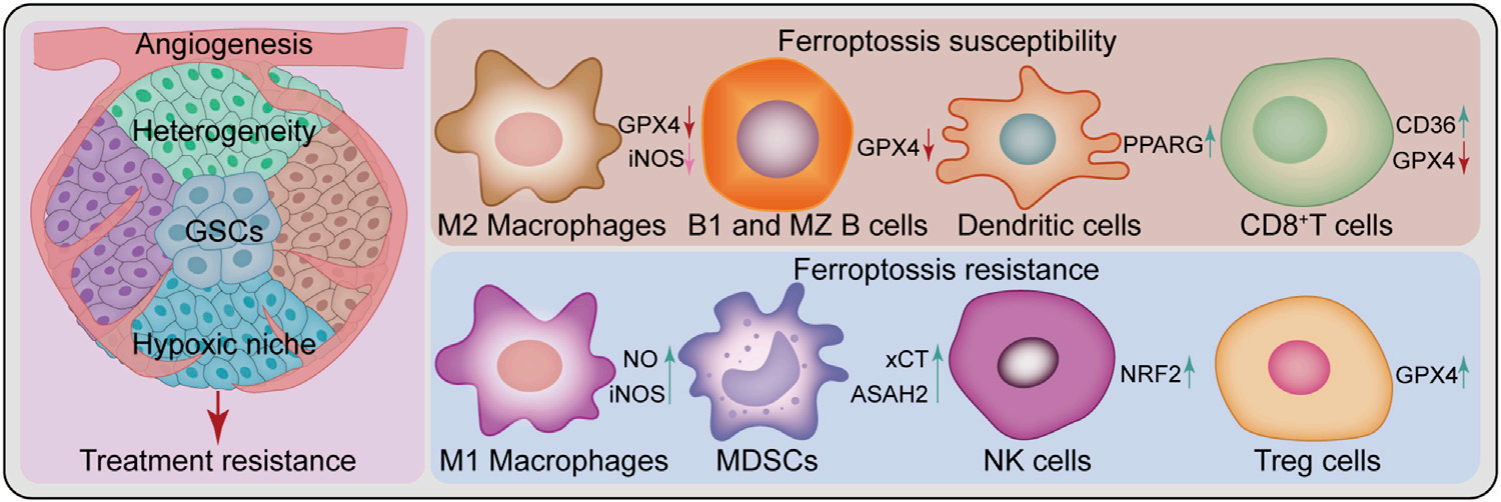
Challenges of ferroptosis in GBM treatment. Ferroptosis in glioblastoma treatment faces several challenges, such as heterogeneity, glioblastoma stem cells (GSCs), angiogenesis, hypoxic niche and a complex immune microenvironment composed of various immune cells. Peritumoral immune cells can be either sensitive or resistant to ferroptosis. Some cell types are sensitive to ferroptosis, such as M2 Macrophages, B1 and marginal zone (MZ) B cells, dendritic cells, and CD8^+^T cells, while others show resistance, such as M1 Macrophages, myeloid-derived suppressor cells (MDSCs), natural killer cells and Treg cells. ASAH2, N-Acylsphingosine Amidohydrolase 2; CD36, CD36 molecule; GPX4, glutathione peroxidase 4; iNOS, inducible nitric oxide (NO) synthase; NRF2, nuclear factor E2-related factor 2; PPARG, peroxisome proliferator-activated receptor gamma; xCT, SLC7A11, solute carrier family 7 member 11.

Broad tumor heterogeneity is a feature of GBM, including genetic, epigenetic, and environmental heterogeneity. Besides inter-tumoral heterogeneity, there is spatial and temporal intra-tumoral heterogeneity, which is considered a key determinant of GBM treatment failure (Patel et al., 2014; Jacob et al., 2020). Furthermore, the presence of GSCs contributes to treatment failure and disease progression for this lethal tumor (Osuka et al., 2021). Meanwhile, these two factors, along with angiogenesis and the hypoxic niche, contribute to treatment resistance (Cheng et al., 2013; Hubert et al., 2016) (Figure 3). Nevertheless, ferroptosis studies based on 2-D cell culture using only a few classical GBM cell lines do not sufficiently reflect the complex tumor heterogeneity and stem cell characteristics. In this regard, induction or inhibition of ferroptosis can hinder GBM growth; however, this is a one-sided argument. In the future, additional studies should be performed under the premise of fully understanding tumor heterogeneity and stem cell characteristics. Novel therapeutic strategies for “State Selective Lethality”, as proposed by James G et al. (Nicholson and Fine, 2021), could provide an opportunity to address this issue. This strategy could render drug discovery and precision therapy that target ferroptosis in GBM more feasible, by inducing or inhibiting ferroptosis in GBM cells to “trap” them in a state that increases their susceptibility to specific treatments.

### 5.2 The complex tumor immune microenvironment

Ferroptosis appears to play a dual role in the TME, causing changes in the function and viability of immune infiltrating cells. The resulting balance between immune evasion and immune elimination directly affects the efficacy of immunotherapy (Xu H. et al., 2021). Differential responses of antitumor T cells to ferroptosis (Drijvers et al., 2021; Ma et al., 2021), GPX4 protects Treg cell survival (Xu C. et al., 2021), differential resistance to ferroptosis of M1 and M2 macrophages and changes in their polarization state (Kapralov et al., 2020; Li and Rong, 2020; Wan et al., 2020), additionally including myeloid-derived suppressor cells (MDSCs) (Zhu et al., 2021), natural killer (NK) cells (Poznanski et al., 2021), dendritic cells (Han et al., 2021), and B cells (Muri et al., 2019) are withal linked to ferroptosis (Figure 3). These current findings demonstrate complex and variable ferroptosis-based crosstalk among individual cells in the TME. In GBM, tumor cells cooperate with peritumoral cells to promote angiogenesis, tumor proliferation, immunosuppression, and brain invasion via multiple communication modes, thereby forming an immune microenvironment conducive to aggressive tumor growth (Broekman et al., 2018). Interactions between immune cells and cancer cells drive GBM transition to a mesenchymal-like state (Hara et al., 2021). As previously mentioned, various immune cells in GBM are involved in the regulation of ferroptosis (Yee et al., 2020; Liu et al., 2022). Nonetheless, the relationship between various immune cells and ferroptosis in their TME remains unknown. Future research should investigate the complex TME cell-to-cell interactions based on ferroptosis and develop strategies to exploit the immunogenic potential of ferroptosis with respect to FINs or inhibitors. Besides, GBM type sensitive to combined ferroptosis modulation and immunotherapy should be identified for effective individualized treatment.

### 5.3 Toxic side effects of ferroptosis

The toxic side effects of ferroptosis are another significant hurdle. Ferroptotic-induced neuronal death and the associated side effects of peripheral nervous system disease, neurodegeneration, and cognitive impairment have been described in a previous review study (Dahlmanns et al., 2021). In addition to chronic injury, acute toxic side effects caused by ferroptosis deserve high attention, including causing brain cytokine storm and necroinflammation, eventually resulting in irreversible brain edema, brain dysfunction, and death. Necrosis is one of the primary manifestations of poor prognosis in GBM and is closely related to thrombosis, hypoxia, and GSCs (Tehrani et al., 2008; Papale et al., 2020). Previous review has highlighted that the effect of treatment-induced necrosis on the CNS is a major clinical challenge in neuro-oncology (Winter et al., 2019). There is a strong relationship between ferroptosis and necroinflammation or neuroinflammation (Friedmann Angeli et al., 2014; Ingold et al., 2018). It should be noted that some subtypes of GBMs possess ferroptosis features. Necrosis in GBM was previously associated with neutrophil-triggered ferroptosis. Moreover, intratumoral GPX4 overexpression or ACSL4 depletion reduced tumor necrosis and invasiveness (Yee et al., 2020). Following the principle of fundamental importance, it is worth considering whether inhibiting ferroptosis prevents tumor necrosis from causing a cascade of irreversible toxic and side effects, thereby benefiting GBM patients. Whether certain methods (such as avoiding thrombosis and improving hypoxia, etc.) can effectively treat GBM by promoting ferroptosis while reducing the occurrence of therapeutic necrosis remains a mystery. Therefore, additional studies are urgently required to address these unanswered questions.

## 6 Conclusions and perspectives

Accumulating evidence suggests that ferroptosis plays a pivotal role in tumor biology and therapy. Moreover, its role is complex and highly context-dependent. Over the past decade, several studies have investigated the role of ferroptosis in cancer. It is expected that further research will unravel deeper regulatory mechanisms of ferroptosis and provide ideas for developing ferroptosis-based strategies for the prevention, diagnosis, and treatment of cancer. Here, we comprehensively describe the role of ferroptosis in the treatment of GBM. The opportunities and challenges facing its clinical application. The data presented here lays the foundation for future basic and clinical research on ferroptosis in GBM. This review focused on ferroptosis peroxidation pathway in GBM therapy. Given the important role of iron homeostasis in ferroptosis and cancer therapy (Brown et al., 2019; Chen G. Q. et al., 2020), this pathway should be considered when targeting ferroptosis in GBM.

Other factors influence the role of ferroptosis. As ferroptosis is inherently compatible with synthetic lethal strategies (Kinowaki et al., 2021), their relationship should be fully considered in the development of anti-tumor drugs. Another factor affecting the effects of ferroptosis is epigenetic regulation. Some epigenetic modulators have been shown to exert anti-cancer effects by targeting ferroptosis. For example, inhibitors of class I histone deacetylases (HDACs) were found to promote ferroptosis in fibrosarcoma cells and prevent ferroptosis in neurons (Zille et al., 2019). A clinical trial (NCT03127514) revealed that HDAC inhibitor sodium phenylbutyrate combined with taurursodiol induced amyotrophic lateral sclerosis in patients (Paganoni et al., 2020). This suggests that HDACs inhibitors may induce ferroptosis in GBM without causing neurotoxic side effects. However, this needs to be clarified experimentally in future studies.

Overall, ferroptosis-based therapy is an emerging clinical intervention with the potential to overcome resistance to currently used treatments such as immunotherapy. Considering that evidence supporting the clinical benefit of ferroptosis-based therapy has been generated from preclinical studies, there is a need for clinical trials to test efficacy and safety of such therapy in GBM patients. Future studies should incorporate organoid model systems, nanotechnology, single-cell sequencing, and spatial transcriptome sequencing to fully exploit the therapeutic potential of ferroptosis in GBM.
